# Enhancement of the Properties of Hybridizing Epoxy and Nanoclay for Mechanical, Industrial, and Biomedical Applications

**DOI:** 10.3390/polym14030526

**Published:** 2022-01-28

**Authors:** Zainab Fakhri Merzah, Sokina Fakhry, Tyser Gaaz Allami, Nor Yuliana Yuhana, Ahmed Alamiery

**Affiliations:** 1 Institute of Laser for Postgraduate Studies, University of Baghdad, Baghdad 10071, Iraq; zainab.fakhri@ilps.uobaghdad.edu.iq; 2 Al Furat Intermediate School for Girls, Ministry of Education, Babylon 11524, Iraq; sokinafahkry@yahoo.com; 3 Department of Chemical and Process Engineering, Faculty of Engineering and Built Environment, Universiti Kebangsaan Malaysia, Bangi 43600, Selangor Darul Ehsan, Malaysia; yuliana@ukm.edu.my (N.Y.Y.); dr.ahmed1975@ukm.edu.my (A.A.)

**Keywords:** nanoclay, nanocomposites, mechanical properties, impact properties, hardness

## Abstract

The strong demand for plastic and polymeric materials continues to grow year after year, making these industries critical to address sustainability. By functioning as a filler in either a synthetic or natural starch matrix, nanoclay enables significant reductions in the impact of nonbiodegradable materials. The effect of treated nanoclay (NC) loading on the mechanical and morphological properties (EP) of epoxy is investigated in this research. The NC-EP nanocomposites were prepared via casting. The investigation begins with adding NC at concentrations of 1, 2, and 3 weight percent, followed by the effect of acid treatment on the same nanocomposites. The evaluation is focused on four mechanical tensile strength parameters: Young’s modulus, maximum load, and % elongation. The addition of NC improved the mechanical properties of the four components by 27.2%, 33.38%, 46.98%, and 43.58%, respectively. The acid treatment improved 35.9%, 42.8%, 51.1%, and 83.5%, respectively. These improvements were attributed to NC’s ability to alter the structural morphology as assessed by field emission scanning electron microscopy (FESEM), a tool for analysing the microstructure. FESEM images were used to visualise the interaction between the NC and EP nanocomposites. The dynamic mechanical properties of the hybrid nanocomposites were investigated using storage modulus, loss modulus, and tan(delta). The results have shown that the viscoelastic properties improved as the fraction of NC increased. The overall findings suggest that these nanocomposites could be used in various industrial and biomedical applications.

## 1. Introduction

The nanoclay (NC) nanocomposites are filler-based materials designed to provide superior mechanical performance at a cheap cost; nevertheless, this results in the NC-EP nanocomposites being brittle, resulting in a loss of mechanical strength [1,2,3]. Numerous investigations have demonstrated that NC nanocomposites have an extremely high tensile strength and Young’s modulus [4,5,6]. Clays are divided chemically and morphologically into several classes, including smectite, chlorite, kaolinite, and halloysite [7]. Another advantage of clay is that it is widely available and inexpensive and has a low environmental impact. Clay minerals are increasingly being employed as natural nanomaterials [8], since they have no adverse effect on the environment. The clay’s widespread use is due to the octahedral (Al or Mg) or tetrahedral layers [9].

As a result, NC nanocomposites have found widespread applications in a range of aerospace and mechanical applications [10,11]. NC composites are frequently employed in applications that need a variety of mechanical properties along with desirable characteristics and physical traits such as lightweight [12,13,14]. NC is utilised as a filler in polymer composite systems, such as nanoclay–epoxy (NC-EP) systems [15], to enhance the mechanical properties of the parent polymer [16,17,18]. Pinto et al. [19,20,21,22,23] studied the mechanical characteristics of EP-based nanocomposites after dispersing nanoclay using sonication [19,20,21,22,23] and surfactants. NC-EP systems can fabricate solid and lightweight materials with a variety of advantageous features [24,25]. The effect of NC on microhardness, Young’s modulus, and impact strength was examined in an elastic EP matrix by adding 3 wt.% of NC [26]. However, the approach they provided for producing the solution combination was ineffective in terms of distributing the NC evenly across the EP substrate. This inefficient distribution was investigated, and it was determined that the mixing process formed a substantial micrometre-sized conglomerate. As a result, the chemical nanoscale compatibility between the polymer matrix and the nanofiller was established, and homogenous nanofiller dispersion was inside the polymer matrix. This achievement demonstrates the critical nature of the interface between nanoclay fillers and the polymer matrix and the critical nature of nanoclay dispersion quality [27]. These interrelated properties dictate the morphology of polymer/nanoclay composites and, consequently, their fundamental bulk properties such as strength, elastic modulus, thermal stability, heat distortion temperature, self-healing, shape memory, and gas barrier [28].

Li et al. [29] examined the modulus of EP-nanoclay composites.

Additionally, Ma et al. [30] have shown considerable advancements in the dispersion and adhesion of EP matrix and carbon nanotubes by nanotube functionalisation. Other research [18,31] investigated whether NC is functionalised to become an EP network component, increasing polarity. However, previous research has proven that homogenous dispersion can be achieved by reinforcing EP-based nanocomposites with NC [32,33,34,35].

Epoxy–nanoclay composites are typically used to improve memory. However, the chemical structures of the thermoplastic materials and the switch temperature or glass transition temperature of the thermoset materials [36] influence the shaping of this memory.

The epoxy–nanocomposite, on the other hand, still has drawbacks such as stiffness and recovery stress. Furthermore, some of the filler percentages have become standard materials for reinforcing epoxy–nanocomposites [37]. Nanoclay-based materials are nontoxic, recyclable, environmentally friendly, and carbon neutral [38]. NC has demonstrated a wide range of advanced applications, including automotive, optically transparent materials, drug delivery, coating films, tissue technology, biomimetic materials, aerogels, sensors, three-dimensional (3D) printing, textiles, printed and flexible electronics, medical and healthcare, and scaffolding [39]. Reinforcing nanoclay with epoxy increased the composite’s tensile strength at 7.5 wt.% nanoclay [40].

Previous studies primarily concentrated on thermoplastic materials such as polyurethane [41]. Thermoset is widely used in memory epoxy, which has critical applications in industry and aerospace. The unique thermochemical properties of thermoset are suitable for memory shaping recovery ratio, environmental durability, rapid response, and easy processing and stability [42]. According to Rousseau et al. [43], changing the chemistry of the epoxy could result in memory shaping by relying on changing the phase-transition temperature. They proposed that the failure strain could be attributed to a change in chemical composition or the use of nanocomposites, in which the glass temperature decreased linearly from 124 to 60 °C, depending on the contents. Aside from the effect of changing glass temperature, chemical and physical crosslinking was discovered to have excellent mechanical properties of high strain and recovery stress [36].

The filler of TiNi macrowires, for example, has demonstrated a significant increase in stiffness accompanied by a higher temperature than the neat epoxy. The benefits of the filler include reshaping memory or either accelerating the increase in temperature or decreasing the specimen’s thinness. Recent research has revealed additional applications for nanoclay-added composites. These applications have suggested that the enrichment in the interfacial bonding between the matrix and reinforcement materials resulted in improved properties. Furthermore, the nanoclay increases the natural fibre’s flexibility and rigidity in a single step [44].

A recent article examined the dynamic mechanical and free vibration properties of various ratios of untreated and treated jute fibre with nanoclay using compression moulding. The results indicate that NaOH concentration and the amount of nanoclay affect the storage and loss modulus, damping factor, and natural frequency. The glass’s temperature increases as the natural frequency increases. Composites comprising 5% treated fibre and 5% nanoclay were shown to have low-strength structural applications in the construction and vehicle industries [45]. According to the literature review, nanoclay/natural fibres hybrid nanocomposites have piqued the interest of researchers due to their improved properties such as mechanical properties, barrier properties, thermal and fire performance. However, there has been little research on the effect of nanoclay-modified epoxy on hybrid composites reinforced with two types of natural fibre materials [46].

The viscoelastic and damping properties of polymer composite materials (sometimes referred to as dynamic mechanical analysis) are advantageous. Additionally, dynamic parameters provide information on interfacial bonding [47], crosslinking density [48], and phase transition [49]. The hybrid composite material significantly improves the dynamic mechanical properties of hybrid-reinforced composites, particularly those reinforced with natural fibres. A study of phenolic hybrid composites of kenaf/pineapple leaf fibres demonstrated an increase in storage modulus over the clean sample [50]. Another work on polyester composites reinforced with snake grass fibres demonstrated balancing storage modulus and damping. The current research evaluates several different forms of nanoclay epoxy composites. The addition of nanofiller to macromolecular polymer chains appears to change their relaxing behaviour [51]. Additionally, it was revealed that inserting nanoclay increased banana fibre’s storage and loss modulus on nanoclay while reducing the loss factor, referred to as tan delta [52]. According to the literature, NC-epoxy hybrids have gained potential interest due to improvements in dynamic and mechanical properties such as barrier properties and thermal and fire performance. To date, however, there has been little research published on the effect of nanoclay-modified epoxy on hybrid composites reinforced with two types of mat form natural fibres [46].

This study aims to increase the amount of NC in the dispersion and improve its uniformity without employing a solvent. Increases in the NC content of the EP matrix resulted in noticeable improvements in the mechanical characteristics of tensile strength and hardness.

## 2. Materials and Methods

### 2.1. Materials

The primary materials used in this report are sulfuric acid and NC. The sulfuric acid was purchased with 95–98% purity from Sigma-Aldrich in Saint Louis, MO, USA. Natural Nano, Inc. provided NC (molecular weight 98.08 g/mole) (New York, NY, USA). Al_2_Si_2_O_5_ (OH)_4_.nH_2_O (0.59) is its chemical formula, and its compositions are O:SiO_2_ (61.19), Al:Al_2_O_3_ (18.11), and Si:SiO_2_ (20.11). Surface area (65 m^2^/g), pore volume (1.25 mL/g), density (2540 kg/m^3^), and refractive index are among the physical properties (1.54).

### 2.2. Treated NC

NC was acid-treated according to a technique described by Biswas et al. [53]. The technique begins with 15 g of NC being treated with 100 mL of 3M sulfuric acid. The mixture is then divided into four pieces and held at a constant temperature of about 90 °C in a water bath. Each sample (portion) is stirred for 3 h at a speed of 200 rpm using a stirrer machine at 3000 rpm for 10 min, and a centrifugal machine separates the paste from the solution. The paste was removed four times with distilled water and dried in an oven at 70 °C for 12 h. The final step is to use a mortar to grind the dried NC.

### 2.3. Matrix

The method used to prepare the epoxy (EP) consists of 25% hardener and 75% transparent EP resin, with the amount of NC varying while the viscosity varies between 11,000 and 14,000 cps of clear EP resin. Furthermore, a nonmethylene dianiline aromatic amine prepared by Daemyung Chemical Technology (Gwangju, Gyeonggi Province, Korea) for use as a curing catalyst (i.e., Amicure 101) was used to obtain an accurate wetting boundary condition for the NC strengthening filler.

### 2.4. Preparation of Nanocomposites (NC-EP)

**Figure 1:**
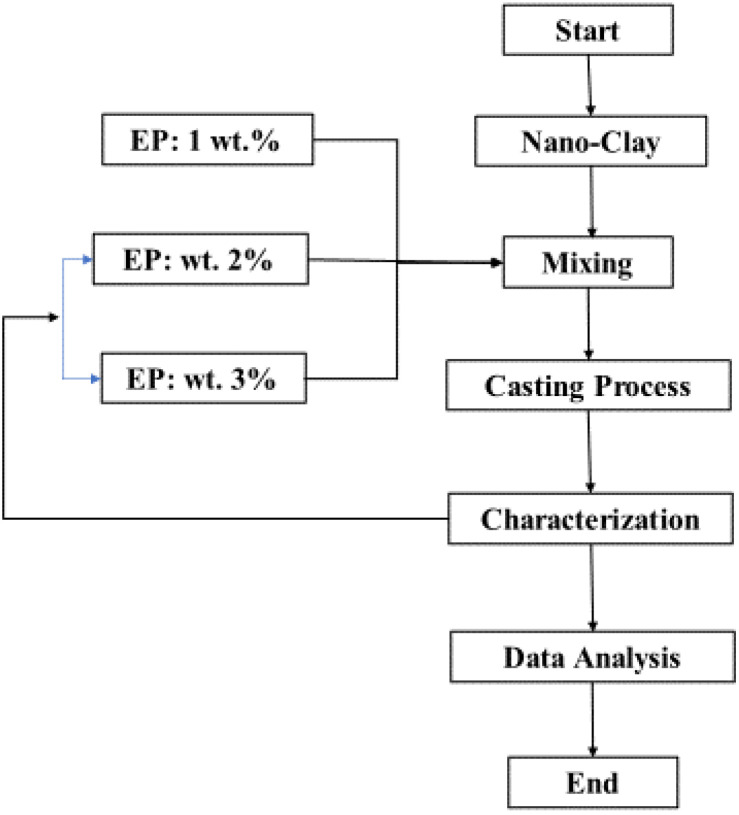
Sample preparation and experimental set-up.

The sample preparation and experimental procedure were carried out following the flow chart depicted in Figure 1. The first step was to make the neat EP by combining 75% resin and 25% hardener to make 100 wt.% EP. The next step was to collect 1 gm of the neat EP and nanoclay. The first mixture was made by combining 99 wt.% (equivalent to 0.01 g) of neat EP with 1 wt.% (equivalent to 0.1 g) of NC. The 2 and 3 wt.% nanoclay were prepared using the same method. Each nanocomposite sample was kept at 25 °C for 10 min before being baked for 3 h at 40 °C to remove any moisture absorbed during the preparation. For 48 h, the reaction between NC and EP was carried out at a room temperature of 25 °C. Table 1 shows the contents of the NC and EP.

## 3. Mechanical Properties

### 3.1. Tensile Properties

**Figure 2:**
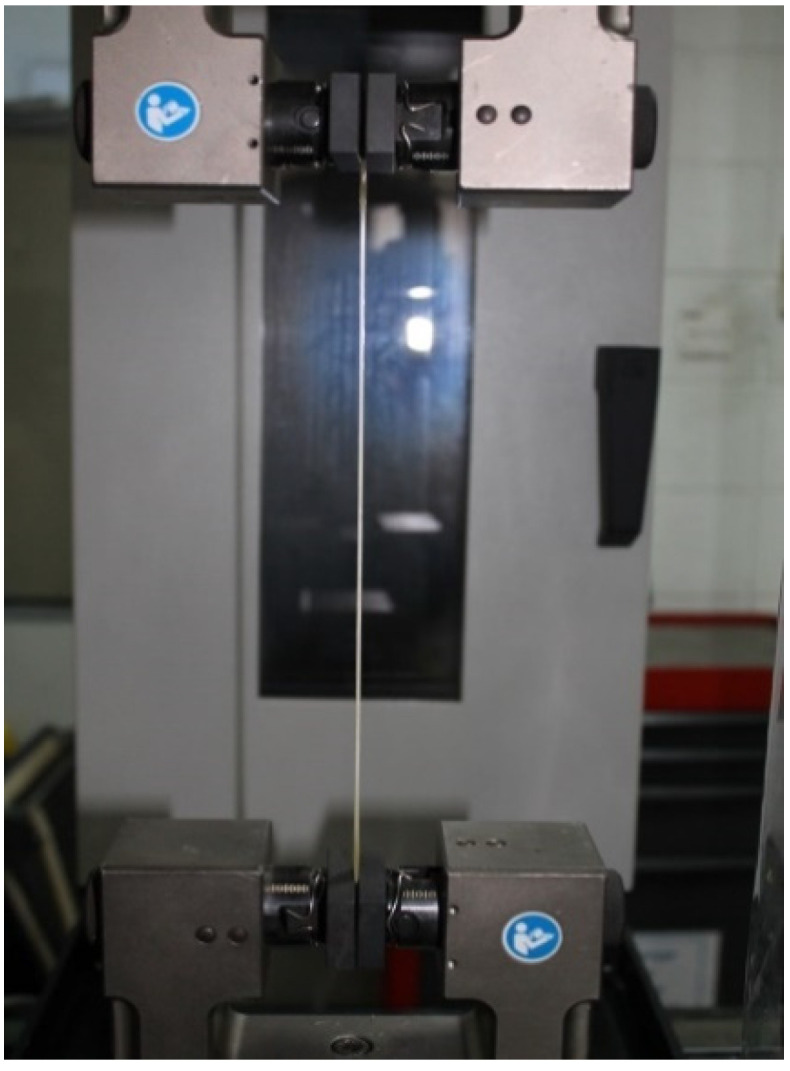
Universal testing machine.

A machine bought from Instron Company (INSTRON 5567, a product of Konigsallee, Düsseldorf, Germany) shown in Figure 2, made dog-bone-shaped specimens for the tensile tests. The American Society set up the Testing and Materials (ASTM) standard D638. The test was used to perform the mechanical ductility test at a temperature of 25 °C and a speed of 1 mm/min. An analysis of flexible and ductile materials was possible because the loading percentage ratio was kept at a low level. In addition, Bluehill software was used to measure the tensile strength, strain, modulus, and percent elongation. Five samples had NC content from 0% to 3% when they were put through the test.

### 3.2. Impact Properties

**Figure 3:**
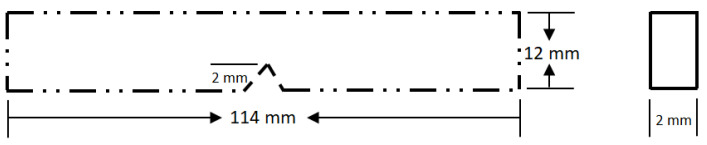
Charpy model impact specimen.

The Charpy impact tests were conducted with a CEAST 6545 pendulum, which was chosen because it meets the expected standard (ASTM D638). Here, 11.4 cm × 1.2 cm × 0.2 cm specimens were put together as a simple beam from both ends. This is shown in Figure 3. The scratch was made to look like a nick or cut at the centre of each sample. For a sample with a single nick in it, this equation [54] was used to figure out the total fracture energy. The calculation was performed according to Equation (1):

(1) $$\left(a_{c}u\right)=\frac{w}{h.b}\times 10^{-3}\;\;\;\;\;\;\;\;\;\;\;\;\;\;\;\;\;\;\;\;\;\;\;\;\;\;\;\;\;\;\;\;\;\;\;\;\;\;\;\;$$

### 3.3. Hardness Properties

**Figure 4:**
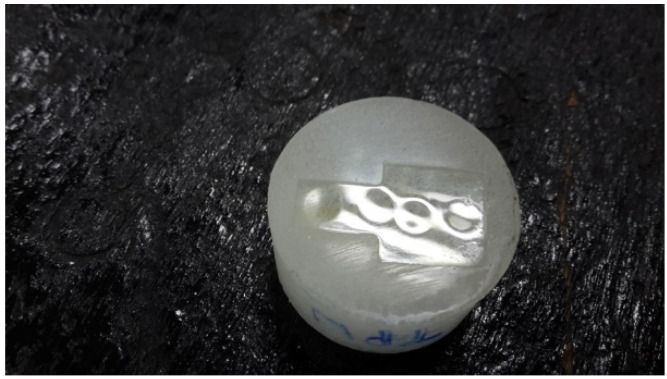
Shore hardness specimen.

In this case, a microhardness tester was used to obtain average hardness readings by measuring two points simultaneously. This procedure shows the setting up of the unit. It was based on Vickers hardness, and the indentation average diagonal length was measured as shown in Figure 4. Equation (2) was used to get the Vickers hardness, as shown in [55]:

(2) $$HV=2Fsin136^{{}^{\circ}}/2/d^{2}\;\;\;\;or\;\;\;HV=1.85F/d^{2}\;\left(approximately\right)$$

## 4. Results and Discussion

### 4.1. Tensile Properties NC-EP Nanocomposites

**Figure 5:**
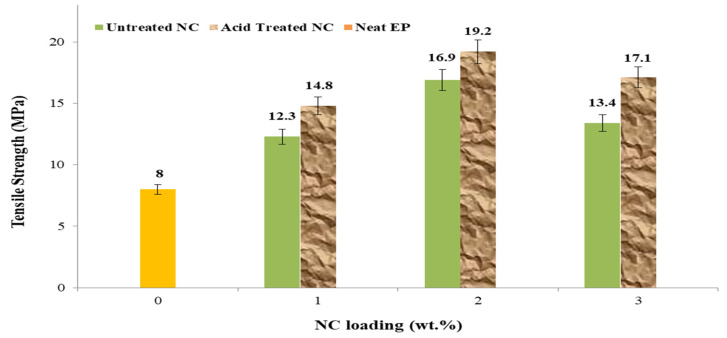
Tensile strength of NC-EP nanocomposites at various contents of NC.

The findings of the untreated EP sample, the untreated EP mixed with 1, 2, and 3 wt.%, and the acid-treated NC mixed with 1, 2, and 3 wt.% are shown in Figure 5. All results regardless of being treated with acid or not have the untreated sample in surplus. The results indicated that the tensile strength decreased when the NC loading increased from 2 to 3 wt.% caused by a slight shift of 1% in the NC loading. The loss in tensile strength at 3% NC loading could result from NC agglomeration, which naturally results in a poor interaction between NC and EP. These findings are consistent with those of Zhang et al. [27].

**Figure 6:**
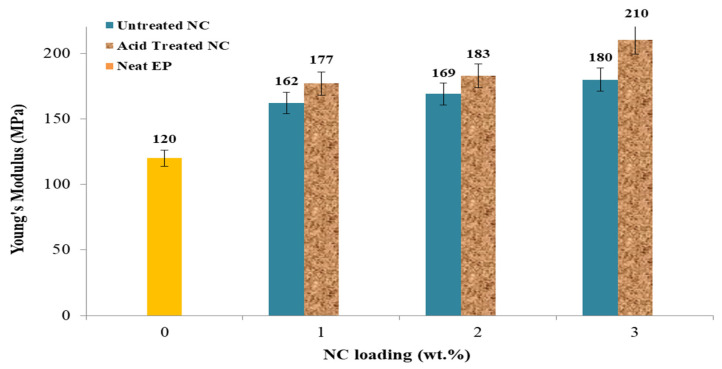
The effect of NC-EP of nanocomposites on Young’s modulus at various NC contents.

Similarly, Figure 6 illustrates Young’s modulus values for the same series of untreated EP samples, untreated EP samples loaded with 1, 2, and 3 wt.% NC, and acid-treated EP samples loaded with 1%, 2%, and 3% NC. Young’s modulus values of 180 and 210 MPa for untreated and acid-treated steels were reported with 3 wt.% NC additives. The increase in Young’s modulus between the two samples is 33.3% and 42.8%, respectively, compared to the untreated EP sample. The effect of acid treatment on the same sample with a 3% NC loading is visible in the 180 and 210 MPa values, representing an increase of around 14%. The findings of loadings of 1 and 2 wt.% NC were nearly identical, with a slight rise in Young’s modulus. The Young’s under nanoclay composite showed the same trend as in case of carbon nanotube. The interpretation relies on the effect of dispersion quality of carbon nanotube colloids [4].

**Figure 7:**
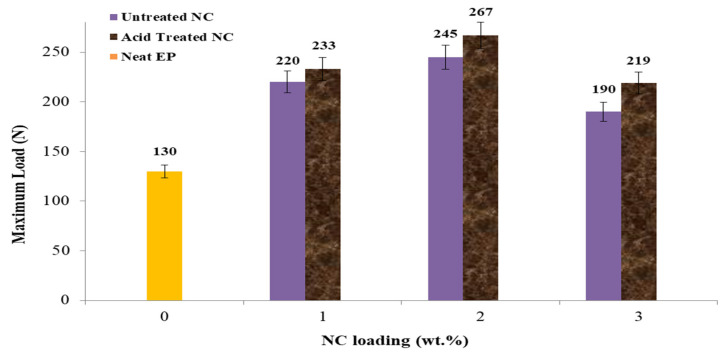
The effect of NC-EP nanocomposites on maximum load at various NC contents.

Figure 7 shows the maximum load for the same sequence. The maximal load of untreated and acid-treated samples attained their maximum values at 2% NC loading, with 245 and 267 N, respectively. These data indicate a maximum improvement of 46.9% and 51.1%, respectively, for the nonacid and acid treatments. Around 8% of the improvement is due to acid treatment. The results indicate that adding NC at a concentration of 3 wt.% reduces the maximum load achieved by the EP-NC sample regardless of the acid treatment.

**Figure 8:**
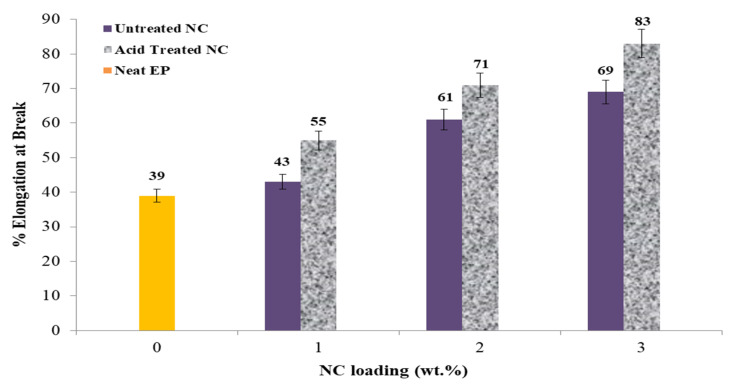
The effect of NC-EP nanocomposites on elongation at break at NC various contents.

The final tensile attribute is the elongation percentage at the break, as seen in Figure 8. For nonacid and acid treatment, the elongation percentages are 46.1% and 68.5%, respectively, at a % -NC loading. The results indicate a growing tendency as the concentration of NC additives increases. As a result, it is impossible to determine if elongation at break hits a maximum or continues to rise following the 3 wt.% -NC addition. The author believes that additional research is necessary to study more significant NC additives.

### 4.2. Impact Properties

**Figure 9:**
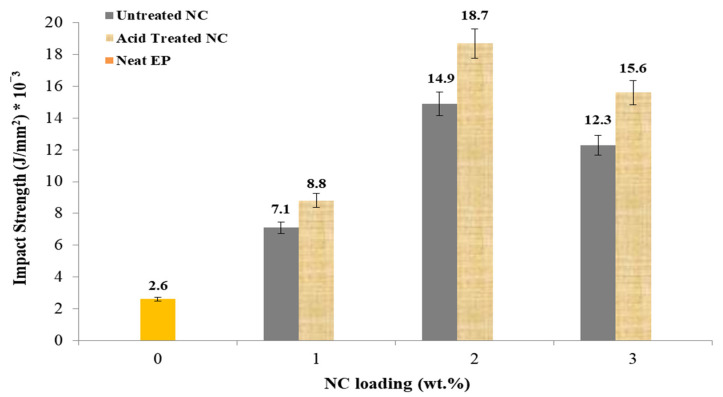
The variation of impact strength with NC content.

Figure 9 depicts the Charpy impact test results for five single-notched samples. The addition of NC at a rate of 2% increased the total gained impact energy from 2.6 to 18.7 J/mm^2^. When the NC content was reduced to 3 wt.%, the impact strength decreased to 15.6 J/mm^2^. The EP nanocomposites matrix combined with NC allowed for the formation of a chemical bond between EP, the filler, and the resin [56].

### 4.3. Hardness Properties

**Figure 10:**
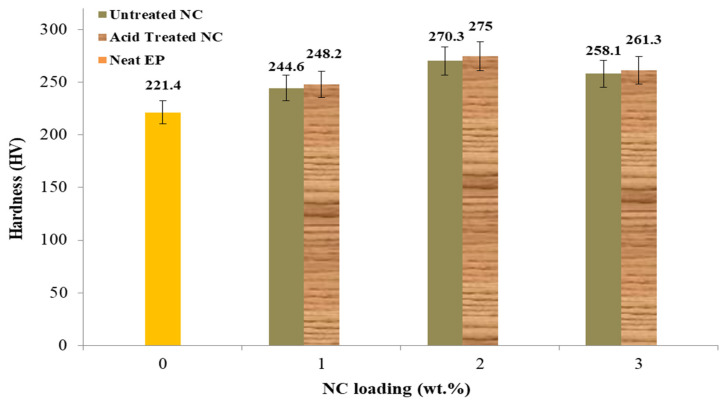
Effects of NC content on nanocomposites hardness.

Figure 10 clearly shows the NC effect on the hardening of the EP matrix, which shows a hardness of 248.2 HV at 1 wt.% NC. By contrast, at NC-2 weight percent, the hardness increased to a maximum of 275 HV. Furthermore, it was discovered that the lowest value of Vickers hardness was found at NC of 3 wt.%. This increase can be attributed to the proposed NC ratio value. The voids produced by the fabrication increased as the NC content increased. Furthermore, the hardness test results at lower temperatures were superior to the sample at room temperature.

### 4.4. Summary of the Mechanical Results

The tensile parameters explored include tensile strength, Young’s modulus, maximum loading, and elongation at break. To completely explore the tensile properties, one sample of untreated NC was used, as well as three samples of untreated NC-EP at 1, 2, and 3 wt.% and acid-treated NC-EP at 1, 2, and 3 wt.%, as shown in Table 2. The most significant relative changes in tensile strength occurred at 2 wt.% EP of both untreated and acid-treated nanocomposites; the results indicate that the highest tensile strength occurred at 2 wt.% untreated NC-EP composites (16.9 MPa; 27.2%) and 2 wt.% sulfuric acid-treated NC-EP nanocomposites (16.9 MPa; 27.2%), (19.2 MPa; 35.9 %). Acid therapy is reported to have an 11.9% effect. However, the improvement was attributable to the NC addition for the acid-treated samples, which permits greater dispersion between the layered silicate of NC and the EP [33]. In terms of Young’ modulus, adding NC at a concentration of 2% to the untreated samples resulted in relative improvements of 33.3% and 42.8%, respectively. Additionally, the acid treatment resulted in a 14.2% improvement. NC 3 wt.% addition resulted in 46.9% and 51.1% reductions in nonacid and acid treatment, respectively, for the maximum load. The effect of the acid treatment resulted in an increase of 8.1% in the maximum load. Finally, the nonacid and acid treatments increased the elongation percentage at the break by 43.5% and 53.0%, respectively, compared to the neat sample. However, the acid treatment resulted in 16.9%.

### 4.5. Field Emission Scanning Electron Microscopy (FESEM)

**Figure 11:**
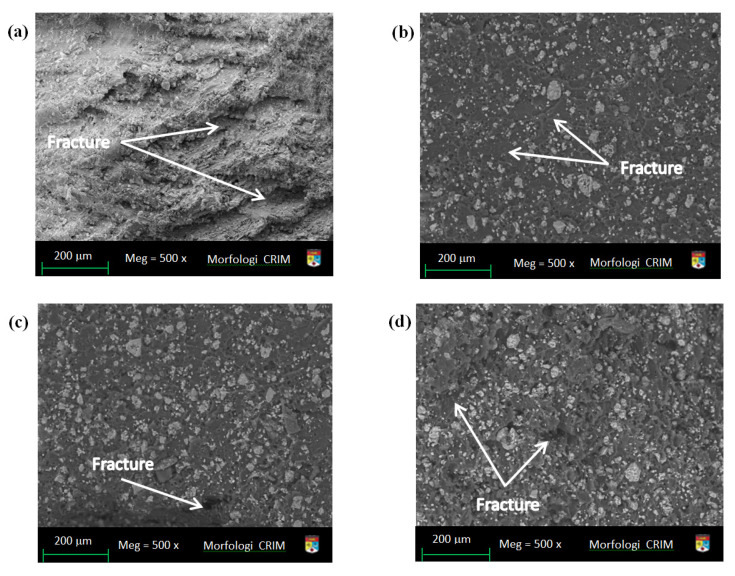
Tensile samples images of FESEM fracture surface (**a**) at pure EP (**b**) at NC content of 1 wt.% (**c**) at NC content of 2 wt.% (**d**) influence fracture surface at NC content of 3 wt.%.

Figure 11 illustrates FESEM images of tensile fracture surfaces made of pure EP resin. As illustrated in Figure 11a, a smooth fracture surface was obtained at a magnification of 500x. The sample containing 1% NC is depicted in Figure 11b, where the fracture surface becomes cloudy and rougher as additional NC is added. Due to the wide specific surface area of the NC grid, the random formation of microfractures indicates that it is an undesirable condition. The inclusion of NC increased the strength of the nanocomposites; however, adding more than 1% of NC could result in the formation of microscale clusters via the NC network. The results indicate that the nanocomposites degrade due to strength transfer from the resin matrix to the NC network. This observation is consistent with those discussed in [57]. Figure 11c illustrates the cross-sectional produced area of the nanocomposites’ fracture surface beyond a tensile test at 2% NC. The FESEM image showed that NC was frequently discontinuous in the matrix [57]. The morphology of the fractured surfaces in the impact test at 3 wt.% NC is illustrated in Figure 11d, indicating that the fracture surface was not uniform. Due to the NC agglomerates, the polymer flow was hampered. The brittleness indication, with a coarser topography, could be detected on the surface of the tidy EP nanocomposites, indicating that the NC pull-out was reduced. The images in the figures have sowed several fractures with varying numbers and sizes.

## 5. Dynamic Mechanical Analysis (DMA)

### 5.1. Storage Modulus (E′)

**Figure 12:**
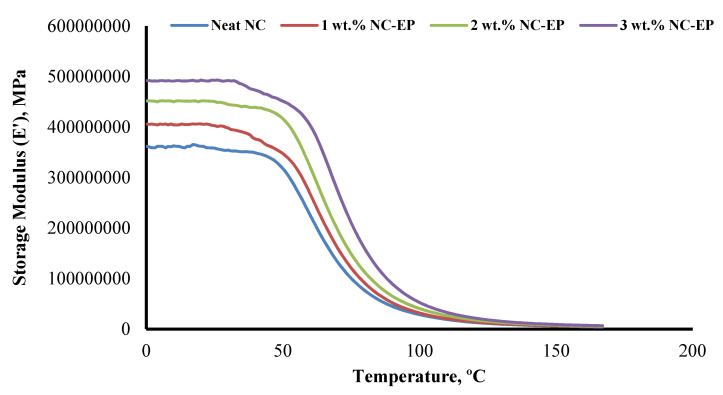
Effect of nanoclay on the storage modulus of the NC-EP at different weight concentrations.

The storage modulus (*E′*) quantifies a material’s elasticity under sinusoidal stress. *E′* provides information on a material’s dynamic mechanical properties, load-bearing capacity [58], crosslink density [59], and fibre-matrix interface strength [60]. Additionally, as illustrated in Figure 12, introducing a nanoclay resulted in a substantial variance in *E*′. The storage modulus of the composites was increased both before and after the glass-transition zone. The glassy region of hybrid composites was improved by 11.2%, 14.4%, and 35.3% with 1, 2, and 3 wt.% NC added to EP, respectively. Additionally, 3 wt.% NC/EP has the best storage capacity, which appears to be attributable to the fibre-reinforced epoxy, which increases the interfacial adhesion strength between fibres and matrix [61].

The *E′* curves in Figure 12 indicated that the composites’ glass-transition (*T_g_*) temperatures were between 44 and 56 °C, indicating that *T_g_* transitions from a glassy to a rubbery state. Thus, the material exhibits stiff and rigid behaviour, as indicated by the material’s high modulus value throughout the mechanical property measurements. Additionally, it is projected that the epoxy matrix’s free volume grows beyond *T_g_* temperature due to the collapsing of the densely packed molecular arrangement.

### 5.2. Loss Modulus

**Figure 13:**
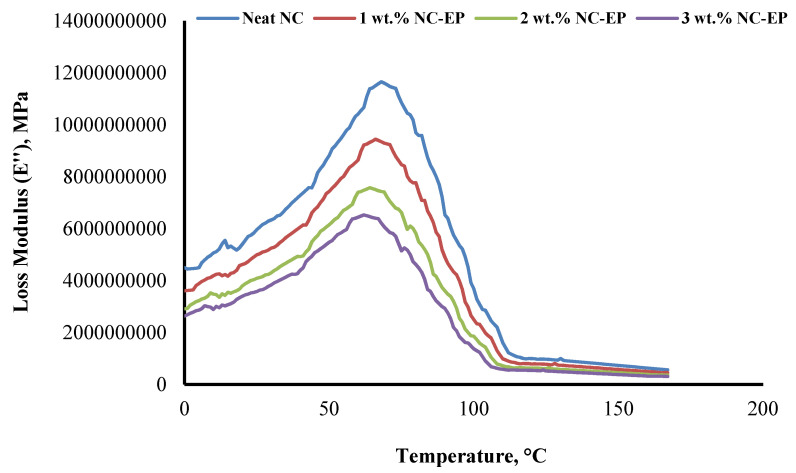
The loss of modulus of EP and the effect of nanoclay on the loss modulus of the hybrid composites.

The loss modulus (*E″*) can be used to analyse the viscous behaviour of materials under oscillating tension [62]. When a material has a large capacity for energy dissipation, it is said to have a high loss modulus, superior damping qualities, and a low damping factor. Thus, materials with a high *E″* value demonstrate a strong potential for industrial applications. Figure 13 illustrates the *E′* behaviour of neat EP and EP composites at weight percentages of NC of 1, 2, and 3 as a function of temperature. All composites reached a maximum peak height between 64 and 72 °C, inside the *T_g_* region. The glassy region’s materials exhibit increased stiffness. However, it becomes stiff and unyielding as the material reaches the glassy temperature. As a result, its loss modulus is small. However, as it passes through the glass transition region and transitions from the glassy to the rubbery state, the material’s viscosity increases dramatically. Similarly to *E′*, when the loss modulus reaches its highest peak height, it exhibits high dissipation energy; however, when the molecules relax, the internal friction decreases, resulting in a decrease in the loss modulus.

### 5.3. Tan(δ)

**Figure 14:**
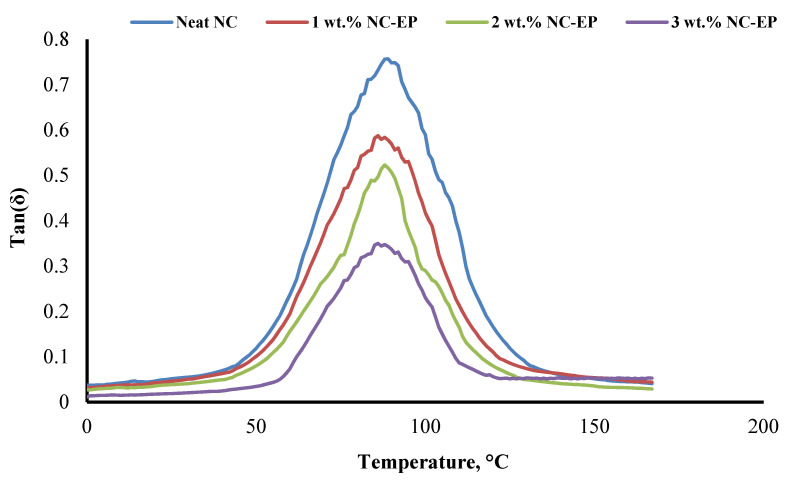
The values of tan delta for EP and the effect of nanoclay on EP.

The ratio of the loss modulus (*E″*) to the storage modulus (*E′*) indicates the loss factor or damping factor. Tan delta is a considerable value, indicating a significant nonelastic strain component. On the other hand, low tan delta values indicate that the material is more elastic. The tan delta curves for EP and the composite sequence of EP-NC at 1, 2, and 3 wt.% are shown in Figure 14. The tan delta peak height of nanoclay composites is lower than that of neat EP, implying that the material has improved damping capabilities due to increased nonelastic deformation and energy dissipation. Additionally, increasing the NC concentration decreases the peak values, indicating that the nanoclay and epoxy are interlocked [61].

### 5.4. Applications

Any material’s application is contingent upon conformity with applicable requirements and standards. When the qualities of the material are enhanced and made modifiable, the material can be used in various applications. In this example, advancements in the qualities of epoxy materials expand their applications, with nanoclay applications divided by kind. Epoxy–NC-based materials could be employed in packaging [63], catalysis [64], and as an additive in the paper-making process [65], as well as medical applications [66]. Additionally, due to the high temperature, it was discovered that it could be utilised as thermal insulation [67]. NC is advantageous in medical applications, particularly vascular grafts, due to its small diameter [68]. NC-epoxy can also be utilised in fire extinguishers [35] and automobile components [69] due to its outstanding thermal stability and tensile strength. Any composite’s applicability is determined by its regulated durability under the conditions of use. Developing biocomposite-based nanoclay poses several obstacles because of the diverse applications of nanoclay, particularly as reinforcements [70]. The sustainability of the environment has a direct impact on economic development. By broadening the application, nanoclay sources improve, benefiting many other sectors [71].

## 6. Conclusions

We investigated the mechanical characteristics of an epoxy–clay nanocomposite. Modified clay boosts the adhesive yield of epoxy under high tensile conditions. The expansion percent to failure of epoxy nanocomposite-1.5% clay is nearly comparable to that of pure epoxy, with a difference of 0.047. Tension strength and failure toughness of epoxy clay nanocomposite are proportional to the amount of nanofiller in the epoxy, and improvement in these parameters is completely evident when using epoxy–clay nanocomposite rather than pure epoxy. Alternatively, adding modified clay to epoxy resin increases the material’s strength and hardness simultaneously. Rack angle, development of new surfaces, and the failure of new piles are all effective methods for enhancing the toughness of an epoxy–clay nanocomposite with intercalation morphology, according to microscopic considerations.

According to reports, NC has been examined for its superior mechanical qualities and high aspect ratio. The current study examines the effects of nanocrystals on EP matrix nanocomposites. The results indicated that adding NC at a rate of 2 wt.% to the EP matrix improved the tensile strength, maximum load, impact strength, and hardness of the nanocomposites when compared to the clean EP matrix. The degree of homogeneity in nanocomposites has a significant effect on their mechanical characteristics. When the NC content was increased from 1 to wt. 3%, the Young’s modulus and elongation at break both rose. Additionally, the impact and tensile strength were enhanced by incorporating NC. The results established the potential benefits of employing NC-EP nanocomposites in a number of applications, including biomedical ones.

The effect of adding nanoclay at concentrations of 1%, 2%, and 3% was investigated on dynamic mechanical characteristics. In comparison to neat EP, the NC-EP composites exhibit extremely high E′ and E″, indicating a strong interfacial connection between the fibres and matrix.

## Figures and Tables

**Table 1:** Ratio of the nanocomposition in terms of EP Resin, NC, and hardeners.

EP	NC (wt.%)	Nanocomposite
EP–Neat	0	NC (0 wt.%)
EP (99 wt.%)	1	NC (1 wt.%)
EP (98 wt.%)	2	NC (2 wt.%)
EP (97 wt.%)	3	NC (3 wt.%)

**Table 2:** Tensile properties of NC-EP nanocomposites.

Sample	Tensile Strength (MPa)	Young’s Modulus (MPa)	Maximum Load (N)	%Elongation at Break
Neat NC	8	120	130	39
1 wt.% untreated NC-EP	12.3	162	220	43
2 wt.% untreated NC-EP	16.9 (27.2%)	169	245 (46.9%)	61
3 wt.% untreated NC-EP	13.4	180 (33.3%)	190	69 (43.5%)
1 wt.% acid-treated NC-EP	14.8	177	233	55
2 wt.% acid-treated NC-EP	19.2 (35.9%)	183	267 (51.1%)	71
3 wt.% acid-treated NC-EP	17.1	210 (42.8%)	219	83 (53.0%)
Effect of acid treatment	11.9%	14.2%	8.1%	16.9%
